# Ionic liquids: a brief history

**DOI:** 10.1007/s12551-018-0419-2

**Published:** 2018-04-26

**Authors:** Tom Welton

**Affiliations:** Department of Chemistry, Imperial College London, Exhibition Road, London, SW7 2AZ England

**Keywords:** Ionic liquid, Green chemistry, Electrochemistry, Synthesis, Catalysis, Industrial applications, Structure, Dynamics

## Abstract

There is no doubt that ionic liquids have become a major subject of study for modern chemistry. We have become used to ever more publications in the field each year, although there is some evidence that this is beginning to plateau at approximately 3500 papers each year. They have been the subject of several major reviews and books, dealing with different applications and aspects of their behaviours. In this article, I will show a little of how interest in ionic liquids grew and developed.

## The beginnings

A brief article such as this cannot cover every aspect of research using ionic liquids and it will inevitably reflect the idiosyncrasies and personal experiences of the author. Even those subjects covered can only receive the briefest mention. If I have left out your contributions to the field, I apologise, no slight is intended. I do attempt to direct the reader to reviews from which they can learn more. Other histories of ionic liquids, with different perspectives, are available in the literature (Wilkes 2002; Angell et al. 2012).

Like the selection of a single source from the many tributaries of a great river, picking a single beginning for a research area is somewhat arbitrary. There are several beginnings to the story of ionic liquids in which they were discovered independently. The earliest of these was when Paul Walden was searching for molten salts that were liquid at temperatures at which he could use his equipment without special adaptations. He discovered that [EtNH_3_][NO_3_] has a melting point of 12 °C (Walden 1914). This was also the first example of a protic ionic liquid (PIL) (Greaves and Drummond 2008; Greaves and Drummond 2015), which were later to become an important sub-class of ionic liquids after being rediscovered by Hiroyuki Ohno (Hirao et al. 2000); Walden’s interest in these molten salts was the relationship between their molecular size and their conductivity. Unfortunately, apart from a brief mention in a study of the parachor of some fused salts in 1927 (Sugden and Wilkins 1929), the potential of this breakthrough went unnoticed for a long time.

Nearly 40 years later, another group independently recognised the potential benefits of lowering the melting points of the molten salts with which they were working. Hurley and Weir were mixing 1-alkylpyridinium halides with ‘true inorganic salts’, such as metal halides (Hurley and Weir 1951) to make solutions from which the metals could be electroplated. During this study, they discovered that the 1-ethylpyridinium bromide-aluminium chloride ([C_2_py]Br-AlCl_3_) 2:1 M ratio mixture was liquid at room temperature. They created a phase diagram for this system, containing two eutectics at 1:2 (45 °C) and 2:1 (− 40 °C) molar ratios and a maximum at the 1:1 molar ratio (88 °C), which they attributed to the formation of bromochloroaluminate species in the melt. The electrodeposition of metals remains an important part of ionic liquid research (Zhang and Etzold 2016).

The 2:1 molar ratio of [C_2_py]Br-AlCl_3_ was later picked up by Bob Osteryoung’s group to study the electrochemistry of two iron(II) diimine complexes, ferrocene and hexamethylbenzene at room temperature (Chum et al. 1975) having noted that this had not been possible in molten Na[AlCl_4_] (m. pt. 175 °C) due to decomposition of the solutes. However, they did note the drawback of this ionic liquid as being it is only at this composition that it is liquid at room temperature. This drove the group to seek a system that was liquid at room temperature over a wider range of compositions, namely 1-butylpyridinium chloride-aluminium chloride ([C_4_py]-AlCl_3_) (Robinson and Osteryoung 1979) which they used to study solute electrochemistry and later characterised using Raman spectroscopy (Gale et al. 1978).

In a separate tributary, other low-melting systems with organic cations were being used. George Parshall used [Et_4_N][GeCl_3_] (m. pt. 68 °C) and [Et_4_N][SnCl_3_] (m. pt. 78 °C) as solvents for platinum catalysed hydrogenation reactions (Parshall 1972). While he did not continue with this work, he did inspire John Yoke to return to a previous observation (Yoke et al. 1963) that [Et_3_NH][CuCl_2_] was an ‘oil’ (a word often used by inorganic and organometallic chemists when something that they expected to be a solid was not) at room temperature to investigate a number of ammonium and phosphonium chlorocuprate systems (Axtel et al. 1973). In another series of papers, Warren Ford explored molten tetraalkylammonium tetraalkylborides (Ford et al. 1973) particularly triethylhexylammonium triethylhexylboride, which had the lowest viscosity of these. With remarkable prescience for future interest in ionic liquids, this included the study of their effects on the rates of organic reactions (Ford et al. 1974) and their toxicity and antimicrobial activity (Rosenthal et al. 1975).

Analysis of the references cited in the papers of those working with chloroaluminate ionic liquids and those working on the other systems suggests that these different groups were unaware of each other’s work. This had a number of contributing factors. Those working with the different systems had very different backgrounds and interests; those working on the chloroaluminate systems were mostly electrochemists, while those working with other systems were more concerned with synthesis and catalysis. Also, these systems had not yet been recognised to be part of a unified concept. Finally, in those days before electronic searching of the literature, searches that we now complete in a few minutes would have taken several days of work. The first confluence of these steams of work was in Chuck Hussey’s seminal 1983 review article on *Room Temperature Molten Salt Systems* (Hussey 1983). In this review, we can see that it was the chloroaluminate systems that had attracted on-going interest, with most work focussed on structures, halogenoaluminate equilibria and physical properties and electrochemistry of the ionic liquids and their solutions. This had been maintained by a handful of enthusiasts and their sponsors, who also worked on more well-established higher temperature systems. The chloroaluminate systems were extremely difficult to handle due to their severe sensitivity to water, which required special equipment such as an inert-atmosphere glove box and created a barrier to entry into this part of field.

## The end of the beginning

In the 1980s, interest in ionic liquids began to slowly spread, with new researchers, such as Ken Seddon and myself, who had not previously worked with high temperature molten salts coming into the field. Also, the range of investigations began to broaden.

[EtNH_3_][NO_3_] reappeared in the literature in 1981, in a short note by Evans et al. about the thermodynamics of its solutions of krypton, methane, ethane, and n-butane and particularly “hydrophobic bonding” (Evans et al. 1981). It is interesting to see that in this paper, the authors noted that [EtNH_3_][NO_3_] had the potential to be used as a non-aqueous solvent for biochemical systems and the importance of its network of hydrogen bonds in controlling its solvent properties. This group went on to study the activity of alkaline phosphatase in water-[EtNH_3_][NO_3_] mixtures and found that at lower concentrations, [EtNH_3_][NO_3_] had a beneficial effect on the enzyme’s activity but that at 80% [EtNH_3_][NO_3_] (*v*/*v*), it was inactive (Magnuson et al. 1984).

Colin Poole picked up on this work and recognised the possibility of using [EtNH_3_][NO_3_] as a stationary phase in gas-liquid chromatography (Pacholec et al. 1982). This then led his group to investigate a number of different ionic liquids in this role (Poole et al. 1986), so initiating the study of applications of ionic liquids in analytical chemistry, which has become a highly active part of modern ionic liquids research (Ho et al. 2014), and eventually led to ionic liquids being commercialised as stationary phases for gas chromatography.^1^ In 1988 using the Surface Force Apparatus (SFA) technique, Horn and co-workers showed that [EtNH_3_][NO_3_] formed layers of ions at a charged mica surface (Horn et al. 1988). This important result went unnoticed by the mainstream ionic liquid community for nearly 20 years.

**Fig. 1:**
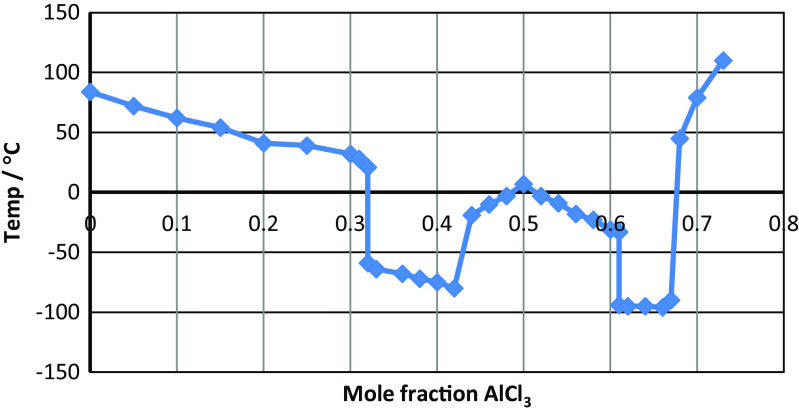
Phase diagram of [C_2_C_1_im]Cl-AlCl_3_

Early in the 1980s, John Wilkes’ group introduced 1,3-dialkylimidazolium cations into ionic liquids for the first time in the form of 1-alkyl-3-methylimidazolium chloride aluminium chloride ionic liquids ([C_n_C_1_im]Cl-AlCl_3_, where *n* = 1–4), with [C_2_C_1_im]^+^ being preferred because it gave ionic liquids with the best transport properties (Wilkes et al. 1982). The [C_2_C_1_im]Cl-AlCl_3_ system was shown to be liquid at room temperature across the composition range from 1:2 to 2:1 mol ratio (Fig. 1). These cations went on to become by far the most popular for making ionic liquids. However, their introduction also led to a controversy over the role of hydrogen bonding in the structures of these ionic liquids. The competing ideas were that interionic interactions in these ionic liquids were either via hydrogen bonding (Tait and Osteryoung 1984) or that they had a stacked structure with anions positioned above and below the plane of the imidazolium ring (Fannin et al. 1984; Dieter et al. 1988). This debate was resolved by recognising that the imidazolium ring protons can indeed act as hydrogen bond donors, but only in the presence of sufficiently strong hydrogen bond acceptors (Lungwitz and Spange 2012; Hunt 2017), and that stacked structures were also present (Elaiwi et al. 1995), particularly for ionic liquids with large anions that are poor hydrogen bond acceptors.

Other interests at this time focused upon the chloroaluminate species present in these ionic liquids (Hussey et al. 1986; Dymek et al. 1988; Abdul-Sada et al. 1989) the impurities arising from their reactions with water (Zawodzinski and Osteryoung 1990; Trulove and Osteryoung 1992) and how these could be removed (Zawodzinski et al. 1987; Abdul-Sada et al. 1987; Noel et al. 1991). The fact that solutions of HCl in acidic compositions (those containing excess AlCl_3_) of these ionic liquids are superacidic (Smith et al. 1989) became of great importance for the application of ionic liquids in the oil refining industry (Harris et al. 2009). Wilkes also reported at this time that acidic compositions could act as a combination of solvent and catalyst for Freidel-Crafts reactions (Boon et al. 1986). The inherent Lewis acidity of these and other ionic liquids has remained an important area for study to this day (Brown et al. 2007).

Interest in other solutes in these ionic liquids was dominated by inorganic chemistry and the coordination chemistry of transition metal species (Hussey 1988).

## The birth of a new field

There is no definition of the concept of a field of research, but there is no doubt that ionic liquids are one. It seems to me that this began sometime at the end of the 1980s and during the 1990s. Undoubtedly, interest in ionic liquids was increasing, but perhaps more importantly by the end of the twentieth century those using different types of ionic liquids and with different academic backgrounds had become aware of each other’s work. Ionic liquids were also being noticed by those who did not work with them.

**Fig. 2:**
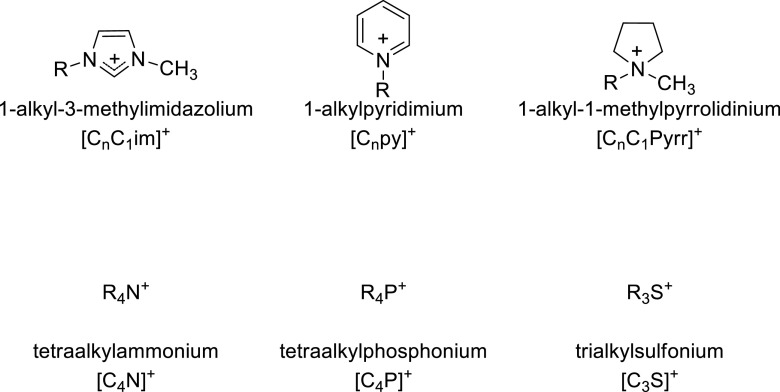
Some commonly used cations for ionic liquids

**Fig. 3:**
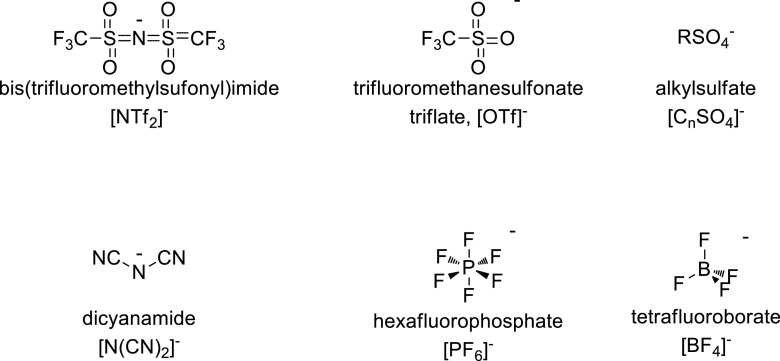
Some commonly used anions for ionic liquids

Another contribution from Wilkes on *Air and water stable 1-ethyl-3-methylimidazolium based ionic liquids* in 1992 is seen by most as ushering a new phase in the development of ionic liquids (Wilkes and Zaworotko 1992), even though the existence of such ionic liquids had been predicted previously (Cooper and Angell 1983) and other air and moisture stable ionic liquids had been in use elsewhere (Poole et al. 1989). The claimed stability of these, thermal as well as hydrolytic, was later shown to be somewhat overplayed (Wang et al. 2017; Maton et al. 2013). Notwithstanding this, these papers do appear to have initiated a period of growth in the number and range of ionic liquids to have been made (Figs. 2 and 3), for example the introduction of the [NTf_2_]^−^ ion (Bonhote et al. 1996), which in turn allowed researchers to broaden the range of cations used (Sun et al. 1997). Phosphonium-based ionic liquids have also become an important sub-class of ionic liquids (Fraser and MacFarlane 2009).

This period also saw a growth in the interest in ionic liquids as solvents for chemical reactions, without necessarily being a component of the reaction itself. This began with a study of Diels-Alder reactions, which are well known to be solvent dependent, in [EtNH_3_][NO_3_] (Jaeger and Tucker 1989). This took a little while to get off the ground and it was a decade later before two papers appeared within a month of each other from the Welton (Fischer et al. 1999) and Seddon (Earle et al. 1999) groups on the use of dialkylimidazolium-based ionic liquids for these reactions. Diels-Alder reactions are also known to be catalysed by Lewis acids, which was exploited in a study of these in chloroaluminate ionic liquids (Lee 1999). Alongside this development, in 1992, the alkylation of sodium p-naphthoxide with benzyl chloride/bromide in molten tetraalkylammonium and phosphonium salts was reported (Badri and Brunet 1992), followed by the room temperature dialkylimidazolium ionic liquids a few years later (Earle et al. 1998).

Probably arising partly from the previous interest of those working with ionic liquids in inorganic solutes as well as its undoubted technological importance, the use of ionic liquids as solvents for transition metal catalysis became a major theme at this time. Throughout the 1980s, Knifton had been investigating hydrogenation (Knifton 1981) and hydroformylation of in a variety of quaternary Group 15 halides (Knifton 1988), although at temperatures that were higher than usually associated with the term ionic liquid.

A key breakthrough using a room temperature ionic liquid came with the nickel catalysed dimerization of alkenes in ethylaluminium chloride containing ionic liquids (Chauvin et al. 1990), which eventually led to the Institut Français du Pétrole’s Difasol process (Plechkova and Seddon 2008). This work was the first to take explicit advantage of the solubility of the catalyst and the insolubility of the reaction products in the ionic liquid. Exploiting this behaviour in biphasic systems became a principal driver for much of the future work in catalysis in ionic liquids (Wasserscheid and Keim 2000; Dupont et al. 2002).

## An explosion of interest—promises made, applications delivered

By the end of the twentieth century, ionic liquids were coming to the attention of a wider audience, e.g. the journalist Michael Freemantle wrote the first of what became a series of reports in *Chemical & Engineering News* (Freemantle 1998). Judging by how often this article is cited in the introductions to papers, his ‘designer solvent’ concept, based on the idea that there are millions of cation-anion combinations that will give ionic liquids from which the ideal ionic liquid for any application can be selected, caught the imagination of many. While no one person was responsible for this, it is true that Seddon was a key figure in advertising the promise that they held. He was convinced that ionic liquids would affect every corner of science and would take every opportunity to tell this to an ever wider audience in, what I used to jokingly call, “The Ken Seddon, ionic liquids are going to save the world roadshow”. He would then follow this up by inviting people to QUILL (the Queen’s University Ionic Liquids Laboratory), the first university–industry collaborative research centre dedicated to ionic liquid research to initiate their research using ionic liquids. He was certainly responsible for bringing many new people to the field.

Also at about this time, Jim Davis introduced the term Task Specific Ionic Liquid (Wierzbicki and Davis 2000; Visser et al. 2001). These ionic liquids differed from the concept of ionic liquids as ‘designer solvents’ (Freemantle 1998) by containing a covalently tethered functional group to one or both of the ions of an otherwise ordinary ionic liquid to imbue the resulting salt with a capacity to interact with dissolved substrates in specific ways (Davis 2004). These ionic liquids have been studied for their potential for a wide range of applications (Lee 2006; Sawant et al. 2011).

The growth in the number of participants in the ionic liquid field was supported by another important change. In 1999, ionic liquids became commercially available in high qualities and at accessible prices from Solvent Innovation GmbH (SI), a company founded by Claus Hilgers and Peter Wasserscheid as a supplier of ionic liquids. While ionic liquids had been available commercially before this, the quality and price of Solvent Innovation’s products made ionic liquids accessible to researchers in quantities that they could make use of. Other suppliers came into the market in the following years. This meant that you did not need to be an expert in the synthesis of ionic liquids in order to be able to use these in your research. I also hope that I played a small part in this growth by writing a review article that summarised the field up to 1999 (Welton 1999). These together with the work of many others led to the well-known explosion of interest in the potential applications of ionic liquids. It is impossible to mention all of their potential applications in a paper such as this, but there are some that should be included.

Probably the first and largest of these was the application of ionic liquids to ‘clean’ and/or ‘green’ technologies and specifically the growing green chemistry movement. The most vocal advocates of ionic liquids as green solvents at this time were Ken Seddon (Seddon 1997) and Robin Rogers (Huddleston et al. 1998) who led a NATO Advanced Research Workshop, on *Green Industrial Applications of Ionic Liquids* held in April 2000 in Heraklion, Crete (together with Sergei Volkov) (Rogers et al. 2003) and then 1 year later a symposium on *Green (or Greener) Industrial Applications of Ionic Liquids* held during the ACS National Meeting in San Diego (Rogers and Seddon 2002). The claim to greenness for ionic liquids at that time rested on their lack of measurable vapour pressure (Earle and Seddon 2000), which was later shown to be untrue for many ionic liquids (Earle et al. 2006). This was an example of a growing problem in the field; results that had been obtained for just a few (sometimes even one) ionic liquids were described as generic properties of all ionic liquids and then later found not to be. Many questioned whether ionic liquids could really be described as green solvents at all (Clark and Tavener 2007; Jessop 2011; Cevasco and Chiappe 2014). My own opinion is that the very concept of a green solvent is somewhat naïve, and we should really be asking whether ionic liquids can contribute to more sustainable production and use of chemicals, which they clearly can do (Welton 2015). Whatever one’s stance in this debate, there is no doubt that this concept brought many rushing into the field, to the extent that the RSC journal *Green Chemistry* was forced to limit the papers about ionic liquids that it would accept (Welton 2011).

A result from early in this period of growth that has proven to inspire many researchers to join the field was the discovery that some ionic liquids could be used to dissolve and regenerate cellulose (Swatloski et al. 2002). This led to a rapid increase in the interest in the potential application of ionic liquids in cellulose processing, but it has transpired that this has been difficult to achieve, and it has not yet been achieved commercially (Gericke et al. 2012; Wang et al. 2012).

This work also led to interest in the use of ionic liquids for the processing of biomass, which has become a major area of activity. At first, this work focussed on using the same ionic liquids that had been identified as capable of dissolving cellulose to dissolve the whole of wood (Kilpelainen et al. 2007), but more recently has included the selective extraction of lignin from the biomass (Brandt et al. 2013). There has also been considerable interest in the production of biomass-derived products from the biomass polymers in ionic liquids (Zhang et al. 2017). This continues to be a highly active area of research and will probably remain so for some time to come.

While most attention this century has been on the application of ionic liquids to organic synthesis (Hallett and Welton 2011), they also have the potential to be useful for inorganic (Freudenmann et al. 2011) and material synthesis (Torimoto et al. 2010).

In 1999, Joan Brennecke caused much excitement when she reported combining ionic liquids with supercritical CO_2_ to form a biphasic system for separations (Blanchard et al. 1999; Jutz et al. 2011). This led to a large amount of work on the solubility of CO_2_ and other gases (Lei et al. 2014) in ionic liquids and eventually to their possible application in carbon capture (Zeng et al. 2017). Of course, the ability of the ionic liquids to act as CO_2_ capture agents can be combined with their ability to be excellent solvents for synthesis and catalysis for the conversion of CO_2_ to higher value products (He et al. 2014; Wang and Wang 2016). Ionic liquids have also been used for electrochemical valorisation of CO_2_ (Alvarez-Guerra et al. 2015). This latter approach is an example of a wider interest in the use of ionic liquids for electrocatalysis (Zhang et al. 2016).

Research into other catalytic reactions in ionic liquids continued apace (Parvulescu and Hardacre 2007; Zhang and Zhang 2011). Perhaps stimulated by the Nobel Prize in the area, many groups worked on the various palladium catalysed coupling reactions (Welton and Smith 2004). It was in this context that the formation of N-heterocyclic carbenes (NHCs) in imidazolium-based ionic liquids first came to our attention (see below) (Mathews et al. 2001). While work continued on liquid-liquid biphasic catalysis, in 2003, an important development was made when two groups applied silica supported ionic liquids in catalytic processes. Christian Mehnert et al. supported their ionic liquids by covalently bonding cations to the silica surface (Mehnert et al. 2002), whereas Peter Wasserscheid, Rasmus Fehrmann and their co-workers introduced their supported ionic liquid-phase (SILP) concept by physisorption (Riisager et al. 2003). The SILP process is not limited to soluble transition metal complex catalysts and can also be used with nanoparticle catalysts, which have also received a great deal of attention (Scholten et al. 2012).

These were not the first examples of supporting an ionic liquid for catalysis (Mehnert 2005). That honour goes to Richard Carlin and Joan Fuller, who incorporated palladium into a gas permeable ionic liquid–polymer gel composed of [C_4_C_1_im][PF_6_]-poly(vinylidene fluoride)–hexafluoropropylene copolymer and used it for the hydrogenation of propene (Carlin and Fuller 1997). The SILP innovation was the development of a practically applicable means to support catalyst solutions of ionic liquids in which the catalyst remains active and stable over extended periods in a continuous gas-phase process.

In the year 2000, the potential of ionic liquids as solvents for enzyme, particularly lipase, catalysed reactions was rediscovered independently by different groups (Cull et al. 2000; Erbeldinger et al. 2000; Lau et al. 2000). This is an example of an area in which it is absolutely not possible to treat all ionic liquids as if they are the same. Some ionic liquids provide a very hostile environment for biocatalysts, whereas others enhance their activities (Weingaertner et al. 2012). This latter possibility led to rapid growth in the area (Kragl et al. 2000; Sheldon et al. 2002) and much subsequent activity on the potential applications of ionic liquids in this technologically important area, with a wide variety of enzyme and reaction types being studied (Itoh 2017). Whole cell biocatalysis, largely in ionic liquid/water biphasic systems (Pfruender et al. 2004), has also been a highly active area for research (Fan et al. 2014).

Other applications rely mainly on the physical nature of ionic liquids, rather than their chemistry. One of these is lubricants. Ionic liquids were first reported as very promising high-performance lubricants to replace other synthetic oils as early as 2001 (Ye et al. 2001). Their non-volatility means that they can be used under vacuum (Liu et al. 2002), which is a real problem for conventional lubricant oils. As well as being used as the lubricant itself, ionic liquids have been used as an additive for conventional lubricants (Phillips and Zabinski 2004). In this role, it is not necessary for the ionic liquids to have a high solubility in the carrier, so they may even be used with quite non-polar lubricants. Finally, they can be applied as thin films (Yu et al. 2006) for use in miniaturised devices. While studying the behaviour of ionic liquids as thin film lubricants, Susan Perkin discovered the remarkable quantized nature of friction in these systems (Smith et al. 2013). Although ionic liquids do show great promise as lubricants, there are still many obstacles to be overcome, such as their thermal and chemical stabilities (Zhou et al. 2009; Zhou and Qu 2017).

It would be remiss to ignore the potential for ionic liquids to make an impact in applications for energy generation and storage (Watanabe et al. 2017; MacFarlane et al. 2014). The possibility of using these as battery electrolytes was one of the first applications considered after the discovery of the chloroaluminate ionic liquids in the second half of the last century (Reynolds and Dymek 1985). However, significant progress was facilitated by the introduction of the less reactive systems later. This coincided with demand for new battery technologies to enable miniaturisation for small devices and length of power output and ease of recharging for transport.

Even the most cursory search of the term lithium battery will quickly find images of these bursting into flames as their flammable organic solvent electrolytes are ignited. Hence, it is not surprising that non-volatile and non-flammable ionic liquids have been proposed as safer replacements for these (Navarra 2013). The advantages of ionic liquids go beyond simple non-flammability to include wide electrochemical windows, stability to the various electrode materials and good discharge ability and cycle ability. Proof of concept performances show that ionic liquids may provide realistic solutions to the conflicting demands of commercial batteries. However, as yet, no ideal ionic liquid system has been developed (Balducci 2017).

Protic ionic liquids have been used for several energy technologies, but as anhydrous proton conductors, they are particularly well suited to fuel cells (Diaz et al. 2014). Again promise is shown, but no ideal ionic liquid has yet been identified. This story repeats for applications of ionic liquids for Dye Sensitised Solar Cells (Zakeeruddin and Graetzel 2009) and supercapacitors (Eftekhari 2017). For all of these applications, the usual advantages of ionic liquids as electrolytes make them attractive, but typical problems associated with ionic liquids, such as high viscosity and disappointing mass and charge transport, still need to be overcome whilst maintaining thermal and electrochemical stabilities. This is likely to require new candidate ions to be developed.

In addition to their use for separations in analytical chemistry, noted above, ionic liquids have found application in separations and extractions for materials as different as those from the nuclear industry (Sun et al. 2012) and bioactive compounds (Ventura et al. 2017). The use of ionic liquids in solid-phase microextraction has shown the potential for commercial application (Ho et al. 2011).

**Scheme 1:**
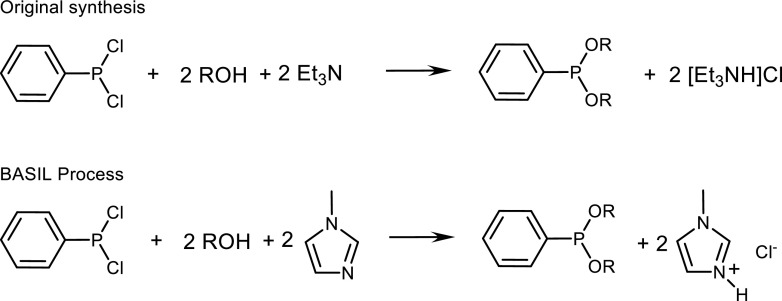
Synthesis of alkoxyphenylphosphanes

Of course, the first commercial application of ionic liquids, BASF’s BASIL (Biphasic Acid Scavenging utilising Ionic Liquids) process, was to solve a separation problem (Volland et al. 2003). BASF used methylimidazole to replace triethylamine as a proton scavenger in the synthesis of alkoxyphenylphosphanes (Scheme 1). This led to a salt by-product, 1-methylimidazolium chloride, with a melting point of 75 °C, which is liquid at the temperature of the reaction and separates spontaneously as a second liquid phase under the reaction conditions. This improvement led to the design of a jet stream reactor for the new all-liquid BASIL™ process, which gave an increased productivity for the process of a factor of 8 × 10^4^ to 690,000 kg m^−3^ h^−1^. At the end of 2004, BASF SE started a dedicated BASIL plant using this technology. The announcement of this success led to a change in the attitude of many sceptics (“ionic liquids will never be used in industry”) as to the practical utility of ionic liquids.

The discovery that ionic liquids could act as entrainers (separation agents) to break azeotropic mixtures to give improved separations by distillation provided another possibility for their use (Jork et al. 2004). While this first example focussed on drying ethanol or THF, many other systems were rapidly added to the list for which ionic liquids could be used (Pereiro et al. 2012). This application combines the ability of ionic liquids to interact more strongly with some compounds rather than others, to favour the separation, with their non-volatility, so that they do not contaminate the distillate. These advantages have also led to ionic liquids being applied for other separations of volatile organic compounds (Salar-Garcia et al. 2017).

An excellent recent example of this is the full production scale plant use of an ionic liquid to remove mercury from natural gas (Abai et al. 2012). The mercury is present in tiny concentrations in the gas stream, but the enormous volume of natural gas production leads to large absolute amounts of mercury passing into the production plant. The combination of a chlorocuprate(II) ionic liquid system with the SILP technology (see above) led to the ability to remove the mercury with these high throughput volumes.

One final application that is generating much interest is the possible application of ionic liquids in pharmaceutical industries. Davis introduced the first ionic liquid derived from an active pharmaceutical ingredient (API) as early as 1998 (Davis et al. 1998). This work has taken two routes: (i) using the kind of ions used for ionic liquids as the counterion of an API that can aid its pharmacokinetics or (ii) two APIs can be delivered as a single ionic liquid (Egorova et al. 2017).

It seems that Ken Seddon and the other advocates of ionic liquids were right and today there are myriad areas of science in which ionic liquids have been applied. They have even found applications in space (Nancarrow and Mohammed 2017)!

## Deeper understanding

While many came to work with ionic liquids due to their exciting applications, others sought a deeper understanding of their behaviours, structures and how these arise (Weingaertner 2008).

Perhaps the first question that is asked about any material is what is its structure. For ionic liquids, this was greatly informed by what was already known for molten salts and the basic fact that opposite charges attract and like charges repel. However, as is the nature of all liquids, this short-range order breaks down at greater distances from any given ion. This was shown very nicely by neutron diffraction studies of [C_1_C_1_im]Cl and [C_1_C_1_im][PF_6_] by Chris Hardacre et al. (2003). This and other studies showed that there were also significant differences in these structures with the [C_1_C_1_im]Cl structure dominated by hydrogen bonding interactions between the chloride ion and the ring protons, while these were largely absent in the [C_1_C_1_im][PF_6_] structure.

**Fig. 4:**
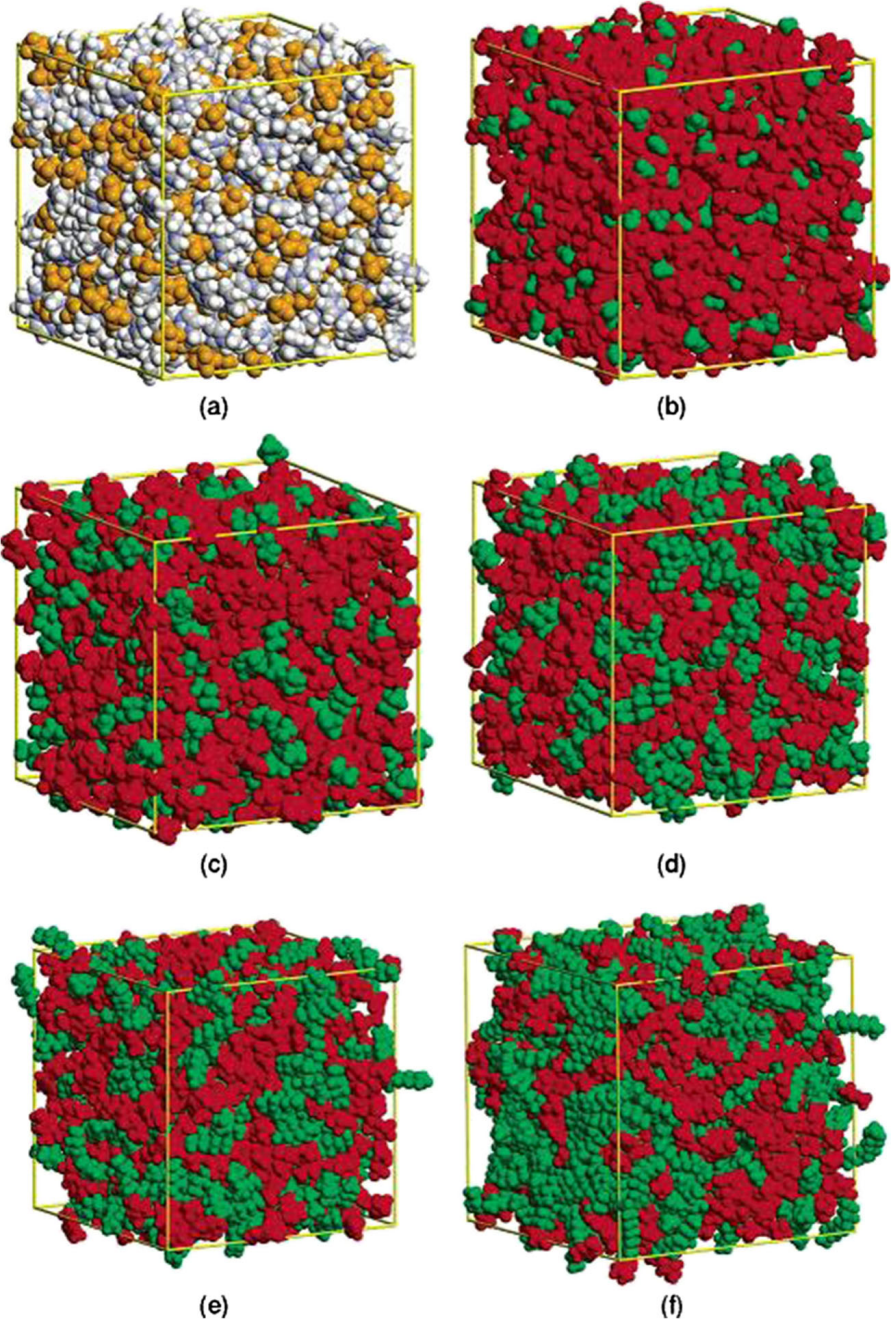
Snapshots of simulation boxes of [C_n_mim][PF_6_]: **a** [C_2_C_1_im][PF_6_] CPK colouring; **b** [C_2_C_1_im][PF_6_] same configuration as in a with red/green (charged/nonpolar) colouring; **c** [C_4_C_1_im][PF_6_]; **d** [C_6_C_1_im][PF_6_]; **e** [C_8_C_1_im][PF_6_]; **f** [C_12_C_1_im][PF_6_]

The next conceptual advance came from a molecular simulation study of [C_n_C_1_im][PF_6_] and [C_n_C_1_im][NTf_2_] ionic liquids in the now-famous ‘Portuguese Flag’ representations (Fig. 4) (Canongia Lopes 2006). These show nanometre-scale structuring in ionic liquids with alkyl side chains longer than or equal to C-4 showing aggregation of the alkyl chains in nonpolar domains, excluded from the charged domains. As the length of the alkyl chain increases, the nonpolar domains become larger and more connected. Depending on the relative size of the charged and non-polar regions, the structures change from a continuous network of ions with isolated non-polar regions to a continuous non-polar phase with isolated islands of charge. These results were shortly afterwards confirmed experimentally (Triolo et al. 2007), although it did take some time before these diffraction results were fully understood (Annapureddy et al. 2010). Of course, not all ions are the same and differentiating between the cations and anions can give further insight that enables rational ion selection to give a range of structures (Shimizu et al. 2010). When the alkyl chains become long enough, ionic liquid crystals can be formed (Goossens et al. 2016). Theory and experiment have moved forward together to grow our understanding of ionic liquid structures (Hayes et al. 2015; Greaves and Drummond 2013; Russina et al. 2017).

This area is an example of a great good fortune that we have experienced, but largely taken for granted, that interest in ionic liquids has developed at the same time that the role that theoretical and computational chemistry can play has been transformed by the power of modern computing. This has led to many advances being theory led. I lack the expertise to review the literature of theory and simulation of ionic liquids, so I will just direct the reader to reviews of quantum chemical (Izgorodina et al. 2017; Hunt 2017), molecular dynamics (Pádua et al. 2007; Dommert et al. 2012), and other methods (Dong et al. 2017).

Understanding how ionic liquid ions interact with each other is key to understanding how these structures arise and how their properties arise from these. These interactions arise from a combination of Coulomb forces, hydrogen bonds, pi-pi interactions, and dispersion forces (Fumino and Ludwig 2014).

Being composed of ions, Coulomb forces are the ubiquitous contributor to the interactions of ionic liquid. Other forces are more dependent upon the structures and chemistries of the constituent ions of the ionic liquids and lead to strong differentiation in their behaviours. From the first debates around hydrogen bonding in ionic liquids (see above), it has been clear that these are sometimes present and sometimes not. For a hydrogen bond to form, the ions must contain both a cation that is a sufficiently strong hydrogen bond donor and an anion that is a suitably strong hydrogen bond acceptor. The ability to change these independently has been noted as a key driver of the designer solvent concept for ionic liquids (Niedermeyer et al. 2013). Consequently there have been many experimental and theoretical investigations of hydrogen bonding in ionic liquids (Dong and Zhang 2012). The doubly ionic nature of cation-anion hydrogen bonds in ionic liquids has proven a particular challenge (Hunt et al. 2015). Dispersion forces also vary with the identity of the constituent ions of the ionic liquids and contribute greatly to the formation of the complex structures described above.

In molecular solvents, such strong cation-anion interactions as are found in ionic liquids often lead to the formation of ion pairs (Marcus and Hefter 2006). Whether these form in ionic liquids has been the subject of much debate. Masayoshi Watanabe and co-workers opened up this discussion with their observation that many ionic liquids had conductivities markedly lower than one would expect from the measured diffusivities of their constituent ions (Tokuda et al. 2006). One potential explanation is that not all of the ions are taking part in the conduction processes, because of the concerted motion of ions of different charge in ion pairs or larger clusters (MacFarlane et al. 2009). Another possible explanation is that the ions do not carry unit charge, because hydrogen bonding leads to charge transfer (Hunt et al. 2015). Both experimentalists and theoreticians have investigated this phenomenon in great detail (Hollóczki et al. 2014), with evidence for both explanations. Of course, these are not mutually exclusive and both may be contributing to the observed conductivities.

The question of whether ionic liquids contain ion pairs, or similar, aggregates is an ongoing one. While conductivity results such as those above do point towards at least the possibility ion pairs, other experiments categorically refute their existence (Lui et al. 2011). In a liquid that is entirely composed of ions, with all ions surrounded by counterions and just by random motion some of these being in closer contact with one of their counterions than others, it is not even straight-forward to define an ion pair. Barbara Kirchner and co-workers have used the idea of using the lifetimes of these contacts to define an ion pair and on this basis have found no evidence in molecular dynamics simulations for ion pairing (Kirchner et al. 2015).

As well as interacting with each other, the ions of an ionic liquid can interact with solute species. Interest in quantifying the interactions between these ionic liquids began to emerge as early as the 1980s (Zawodzinski and Osteryoung 1989). Perhaps not surprisingly, interest in the chloroaluminate systems focussed on parameters arising from Lewis acid interactions (Mantz et al. 1997). Elsewhere, Michael Abraham et al. (1991) applied his multiparameter model to the ionic liquids that Poole had been using for his GC columns (Poole et al. 1986). It was shown that the ionic liquids studied were all strongly dipolar and hydrogen bond acceptors. This work was followed by studies of systematically selected ionic liquids, both chromatographically (Anderson et al. 2002) and spectroscopically (Crowhurst et al. 2003). These and other studies showed that ionic liquids are highly polar solvents in terms of their dipolarity/polarizability with a much wider range of hydrogen bond donor (cation) and hydrogen bond acceptor (anion) abilities (Poole 2004). Some studies suggested that the ionic liquids were stronger hydrogen bond donors than others. This apparent discrepancy was explained by the nature of the solute probe, with charged hydrogen bond acceptor solutes reporting higher values than uncharged solutes (Ab Rani et al. 2011). In a study of the effects of ionic liquids on Diels-Alder reactions, it was found that the strength of the hydrogen bond from the ionic liquid cation to a solute was determined by the ability of the cation to donate a hydrogen bond moderated by the ability of the anion to accept a hydrogen bond (Aggarwal et al. 2002a). A derivative of this concept was later used to explain the solubility of cellulose in ionic liquids (Hauru et al. 2012).

It is through their interactions with solutes that ionic liquids affect chemical reactions (Chiappe and Pieraccini 2005). Linear solvation energy relationships (LSERs) have demonstrated the effects of hydrogen bonding and dipolarity/polarizability on the rates (Crowhurst et al. 2006) and selectivities (Bini et al. 2008) of reactions in ionic liquids. For the majority of reactions studied, the LSERs can be achieved using both ionic liquids and molecular solvents, meaning that differences between these are quantitative in nature, rather than qualitative. However, it has been shown that it is sometimes not possible to achieve this, indicating a change in the mechanism of the reaction.

For the reaction between the dimethyl-4-nitrophenylsulfonium ion [(p-NO_2_PhS(CH_3_)_2_]^+^ with the chloride ion in molecular solvents the reaction rate decreased with increasing chloride ion concentration (Hallett et al. 2009), due to a stepwise mechanism via ion pairs. In all of the ionic liquids studied, the rate increased linearly with increasing chloride ion concentration, as one might expect from a simple nucleophilic substitution that does not involve ion pairing. This shows that no ion pairs were formed in the ionic liquid that were sufficiently long-lived or in high enough concentration to be kinetically relevant. This was used to propose that the constituent ions of the solution are highly electrostatically screened.

So far, we have only scratched the surface of understanding how ionic liquid-solute interactions lead to changes in reactivity and this remains an important area of research (Keaveney et al. 2017).

**Fig. 5:**
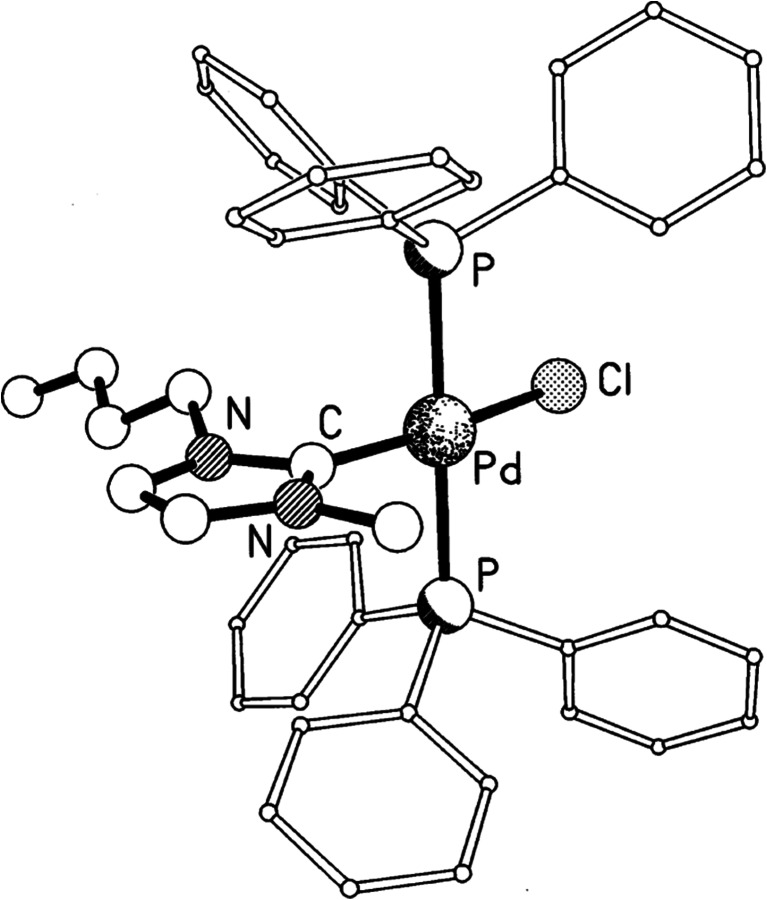
The molecular structure of a mixed phosphine-NHC palladium complex formed in [C_4_C_1_im][BF_4_]

As early as 2001 (Mathews et al. 2001), it had been noted that the addition of palladium to imidazolium-based ionic liquids *could* lead to the formation of compounds containing N-heterocyclic carbene ligands (Fig. 5), but that this did not always occur. In this case, it was necessary to add NaCl to solutions in [C_4_C_1_im][BF_4_] for the complex to form. Soon after, Varinder Aggarwal et al. noted the low product yields in the base-catalysed Baylis-Hillman reaction of methyl acrylate and benzaldehyde in the presence of [C_4_C_1_im]Cl (Aggarwal et al. 2002b). They explained this by a side reaction of the benzaldehyde with [C_4_C_1_im]^+^ via a NHC formed by deprotonation of the imidazolium cation. From these results, interest in this reactivity began to grow (Dupont and Spencer 2004) and it became clear that a sufficiently basic anion was required to deprotonate the imidazolium ring to produce the NHC. As the years progressed, more compounds incorporating NHCs were identified, for example 1,3-dialkylimidazolium-2-carboxylate (Gurau et al. 2011). Sometimes, the NHCs have been shown to be very positive, as in their use to catalyse the Benzoin condensation (Kelemen et al. 2011). At other times, they have been a hindrance, as in their reaction with cellulose during its processing in [C_2_C_1_im][CH_3_CO_2_] (Clough et al. 2015).

The observation of products that could be the result of reactions with NHCs does not prove that these are present in the ionic liquids as free species. These products could arise from concerted reactions of the imidazolium cation, as suggested by a recent computational investigation of the reaction of CO_2_ with [C_2_C_1_im][CH_3_CO_2_] (Yan et al. 2017). Oldamur Hollóczki et al. have also suggested that the carbene trap is required for the reaction to occur, or at least the presence of neutral electrophiles makes the formation of the NHC more likely (Hollóczki et al. 2013). Recent kinetic observations of the Benzoin condensation have given the first strong evidence of the spontaneous formation of NHCs in ionic liquids (Daud et al. 2017). However, whilst this is evidence of the NHC being the catalyst for the reaction, it is not an absolute confirmation of this, because what the result shows is that the C^2^-H bond of the imidazolium ring is not involved in the rate determining step of the reaction, not that the NHC definitely is.

Ionic liquid ions do not only interact with themselves and solute species, they also interact with the materials that they interface with. The importance of applications such as SILP makes understanding the gas-liquid interface particularly important. Hence, a number of techniques have been used to probe this. Generally, it has been found that the cations and anions usually share the surface layer without any particular layering, but that the alkyl chains of the ions tend to aggregate at the surface, with the ion orientated such that the alkyl chain is presented as the surface (Santos and Baldelli 2010). For anions with trifluoromethyl groups, these orient themselves to the surface, provided that the cation alkyl chain is sufficiently short that its ionic head group is not immersed deep in the liquid away from the surface. It has been proposed that the driving force for this behaviour is that the highly charged moieties of the ions are driven to the bulk where possible to avoid the energy cost of nearest neighbour interactions, leaving the uncharged alkyls chains to populate the interface (Lovelock 2012).

The use of ionic liquids in electrochemical applications has led to a great deal of interest in the behaviour of ionic liquids at charged surfaces (Fedorov and Kornyshev 2014). This behaviour has turned out to be complex and is yet to be fully understood. This partly arises from our understanding of electrochemical phenomena being dominated by classical dilute electrolytes, which do not aid the understanding of systems composed entirely of ions.

In 2007, Rob Atkin and Greg Warr published the results of a study that used atomic force microscopy to show the layering of [EtNH_3_][NO_3_], [PrNH_3_][NO_3_], and [C_2_C_1_im][CH_3_CO_2_] on mica, silica, and graphite surfaces (Atkin and Warr 2007). They showed that the degree of layering, up to 8 or 9 layers or 5 nm, for [EtNH_3_][NO_3_] was dependent upon the charge and roughness of the surface and the structure of the ionic liquid. This was shortly followed by an X-ray reflectivity study of the temperature-dependent structures of three ionic liquids with the *tris*(pentafluoroethyl)trifluorophosphate anion at a charged sapphire substrate (Mezger et al. 2008), which also showed the formation of layers of ions. Using SFA, Perkin and co-workers not only confirmed the layering of ions, but also demonstrated that as the alkyl chains of her ionic liquids increased from [C_4_C_1_im][NTf_2_] to [C_6_C_1_im][NTf_2_], the layering changed from simple cation-anion monolayers to tail-to-tail cation bilayers (Perkin et al. 2011). Thinking in 2007 by Alexei Kornyshev (Kornyshev 2007) on the nature of the double-layer and capacitance in ionic liquids gave a theoretical framework in which these results could be conceptualised.

In 2013, Matthew Gebbie et al. (2013) used SFA with [C_4_C_1_im][NTf_2_] to measure attractive forces between gold and mica surfaces extending well beyond the measured surface layering, up to 35 nm. They used these results to argue that ionic liquids should be considered to be dilute electrolytes, with the vast majority of ions being undissociated and so not contributing to the electrostatic screening of the electrodes. This led to immediate controversy (Perkin et al. 2013). While the experimental results are not in doubt, the explanation of these and subsequent confirmations are still a hot topic (Gebbie et al. 2017). My own view is that so far the only type of organisation that has been considered at an electrode is the displacement of ions into surface layers and other forms of ordering should be considered. For example, if the ions do not completely freely rotate in this extended layer and instead became orientated with respect to each other, this could contribute to these longer range forces.

## What is in a name?

Throughout the last century, there was much controversy over which materials should be included as ionic liquids and which should be excluded. Clearly, the term ionic liquid simply means a liquid composed of ions. However, the constraint that ionic liquids should be liquid at temperatures below 100 °C was added around the turn of the century. I often find myself accused of introducing this constraint in my 1999 review (Welton 1999). However, this is most certainly not the case, and let me repeat what I did say there:

“Room-temperature ionic liquid, non-aqueous ionic liquid, molten salt, liquid organic salt, and fused salt have all been used to describe salts in the liquid phase. With the increase in electronic databases, the use of keywords as search tools is becoming ever more important. While authors are free to choose any name that they wish for their systems, I would suggest that they at least include the term ionic liquid in keyword lists. In this paper, I allow the term ionic liquid to imply that the salt is low melting.”

If it was not me, where did this come from? The first time that I was aware that the temperature of 100 °C was associated with ionic liquids was at the NATO Advanced Research Workshop on *Green Industrial Applications of Ionic Liquids* held in April 2000 in Heraklion, Crete. It appears that the purpose of doing this was to make it clear that the meeting was not going to include high temperature inorganic molten salts in its programme. This led to an unfortunate schism between the communities working with these different systems, which has perpetuated to the detriment of both. Also, there is no scientific justification for believing that a salt with a melting point of 90 °C is in any necessary way different from one with 110 °C. I hope that the time has come for this constraint to be dropped.

Strictly speaking, the term ionic liquid implies that the liquid is only composed of ions, with no molecular species present. This does not mean that materials that do have molecular constituents, the nearly ionic liquids, are not of interest.

**Scheme 2:**
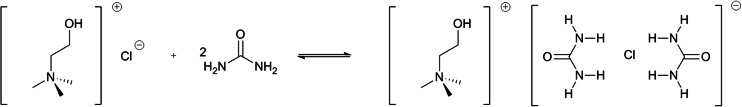
Proposed complex formation in a DES. The ChCl-urea DES has a eutectic point at a 1:2 ratio

The most well-known of these systems are the Deep Eutectic Solvents, which were first introduced by Abbott et al. (2003). The first of these was formed by mixing choline chloride and urea in a 1:2 molar ratio. The concept was that the hydrogen bond between the donor molecule and the chloride ion is so strong that it generates an ‘ion’ which is much larger (Scheme 2), breaking down the cation-anion interactions and lowering the mixture’s melting point, so generating an ionic liquid-like material. It was later shown that this was a somewhat naïve view of the bonding in these systems (Ashworth et al. 2016) with many different types of H-bond formed. For example, the urea is found to form a H-bonded [urea(choline)]^+^ complexed cation in addition to the [Cl(urea)_2_]^−^ ion. Andy Abbott tells me that the name Deep Eutectic Solvent came about because a referee of the first paper was insistent that they could not be called ionic liquids. This does not seem to have done them any long-term harm as they have become an area of a great deal of activity (Zhang et al. 2012).

**Scheme 3:**
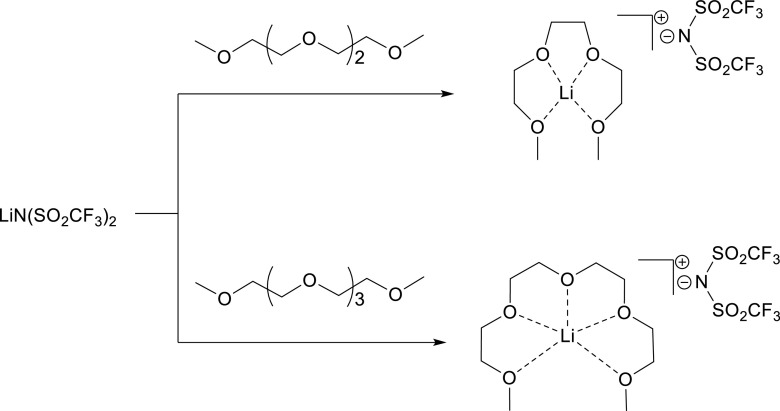
Proposed complex formation in lithium glyme solvate ionic liquid. The lithium salt-glyme molar ratio is 1:1

Another group of materials that have received attention by the ionic liquid community are the lithium-glyme solvate ionic liquids (Scheme 3). These share conceptual similarities with the DESs in that a complexing material, this time a glyme to a lithium salt, is added to make a complex ion that is larger, so breaking down cation-anion interactions and reducing the melting point of the mixture.

These were first introduced by Watanabe and co-workers for use as electrolytes for lithium-ion batteries (Ueno et al. 2012), for which they are gaining much attention (Watanabe et al. 2017). They recognised from the beginning that while long-lived [Li(glyme)]^+^ complex cations are formed, other species are possible and that the precise composition depended upon competitive interactions between the glymes and the Li^+^ cations and between the counter anions and the Li^+^ cations. These glyme-based ionic liquids are related to a much older material, 5 M lithium perchlorate-diethyl ether, which although no longer used, was used as a solvent for organic synthesis for some time (Heydari 2002) and was considered to be a ‘fused salt’ containing both [Li(ether)]^+^ and [Li(ether)_2_]^+^ ions (Ekelin and Sillén 1953).

## Where are we now

I started this paper with the metaphor of the history of ionic liquids as separate small streams slowly joining as tributaries of a great river that then flowed its way across the scientific landscape. Now, with the many thousands of people who are studying and researching in that great river, we can see that this is moving on apace and there is much to still be discovered and understood. However, I do notice that another change is underway. It is now rare for individual scientists to be expert, or even interested, across the whole field. Meetings on ionic liquids are most often now focussed on one or two of their applications, or a subset of their physical properties and that great river is separating into separate channels as it flows through its delta. This is probably to be expected of a field of study that grows to this size and I consider myself very lucky to have experienced so much of this and to have contributed to it.

## Footnotes

1 Supelco Ionic Liquid GC Columns. https://www.sigmaaldrich.com/analytical-chromatography/gas-chromatography/columns/ionic-liquid-literature.html
